# Sex Comparisons for Very Short-Term Dynamic Constant External Resistance Training

**DOI:** 10.3390/jfmk3040050

**Published:** 2018-10-18

**Authors:** M. Travis Byrd, Taylor K. Dinyer, Haley C. Bergstrom

**Affiliations:** Department of Kinesiology and Health Promotion, University of Kentucky, Lexington, KY 40502, USA

**Keywords:** dynamic constant external resistance (DCER), very short-term resistance training (VST), bench press, bench throw, gender

## Abstract

This study compared sex responses for strength and barbell velocity from very short-term resistance training (VST, consisting of 2–3 training sessions) for an upper body dynamic constant external resistance (DCER) exercise (bench press [BP]). Ten females (mean ± standard deviation (SD) age: 21.3 ± 3 years, height: 166.2 ± 6 cm, body mass: 71.4 ± 10.7 kg) and 10 males (mean ± SD age: 24.6 ± 4 years, height: 179.5 ± 8 cm, body mass: 88.6 ± 11 kg) completed a pre-test visit to determine the BP 1 repetition maximum (1RM) as well as the mean (BP_MV_) and peak (BP_PV_) barbell velocities from the BP 1RM. The VST involved three training visits where the participants performed 5 sets of 6 repetitions, at 65% of the 1RM. The post-test followed the same procedures as the pre-test visit. There were significant increases in 1RM strength for both the males (5.1%) and females (5.4%) between pre-test and post-test. There were no significance differences between sex for mean (BP_MV_) and peak (BP_PV_); however, overall there was a 32.7% increase in BP_MV_ and a 29.8% increase in BP_PV_. These findings indicated an increase in strength and barbell velocity for both males and females as a result of VST upper body DCER exercise in untrained subjects.

## 1. Introduction

A common mode of training is dynamic constant external resistance (DCER). This form of resistance training is used in injury rehabilitation and for general fitness as well as sports performance development to increase strength in sedentary, active, and highly trained individuals [1]. Specifically, for untrained males, DCER training utilizing the bench press, performed at the 7 repetition maximum (RM) for 1 or 3 sets, resulted in an increase of 9.2 ± 3.9% and 10.1 ± 5.2% respectively, in pre- to post-training 1RM measures after 6 weeks of training [2]. Typically, studies examine training protocols consisting of 18 to 36 training sessions within a 6- to 12-week period [2,3]. 

Skeletal muscle and performance adaptations to resistance training exercise programs are well documented [4]. Resistance training results in both neural and skeletal muscle adaptations [5,6]. Resistance training has been shown to increase movement velocity, causing a shift in the force-velocity curve, resulting in increased rate of force development, which has been suggested to reflect neuromuscular adaptations [7]. During the first few weeks of training, a significant increase in strength may be attributed to neural adaptations, such as an increased central nervous system (CNS) efferent neuron activity [5,6,8]. The effects of skeletal muscle hypertrophy on strength become more dominant after 8 to 12 weeks of resistance training [9].

The very short-term resistance training (VST) model utilizes 2–3 training sessions to determine the minimal number of sessions necessary to observe the early skeletal muscle and performance adaptations. Previous VST studies have examined the effects on forearm flexor [10] and knee extensor performance [11,12] using isokinetic forms of training, and has recently been applied to both lower body [13,14] and upper body [15] DCER exercises, as well as the efficacy of creatine supplementation to increase strength [16].

Traylor et al. [10] showed a sex difference in response to the VST model for isokinetic exercise. Specifically, there were performance increases pre- to post-test for the male participants, but not for the female participants [10]. However, other researchers utilizing the VST model have demonstrated peak torque increases for female subjects ranging from 11.5% to 40.2%, depending on the training velocity [11]. Currently, it is unknown if there are sex differences in response to VST utilizing a DCER exercise. In addition, there is limited research regarding the applicability of the VST model to DCER training, which is more widely used by athletes as well as recreational lifters. Therefore, the purpose of this study was to compare sex responses for strength and barbell velocity from VST upper body DCER training (bench press [BP]).

## 2. Materials and Methods 

### 2.1. Experimental Design

The study involved 7 visits with 48–72 h between each visit, including signing of the informed consent form, a familiarization visit, pre-test visit, three training visits, and one post-test visit. Thus, each participant completed the study, from pre-test to post-test, within 13 to 19 days. For the pre-test visit, the subject’s 1 repetition maximum (1RM) for the BP was measured as well as the mean (BP_MV_) and the peak (BP_PV_) barbell velocities from the BP 1RM. The mean (BT_MV_) and peak (BT_PV_) velocities were also determined from the barbell bench press throw (BT) test, utilizing 35% of the participant’s BP 1RM as resistance. Visits four through six were the three training visits. Visit seven, the post-test, followed the same procedures as the pre-test visit. 

### 2.2. Participants

Ten female (mean ± standard deviation (SD) age: 21.3 ± 3 years, height: 166.2 ± 6 cm, body mass: 71.4 ± 10.7 kg) and 10 male (mean ± SD age: 24.6 ± 4 years, height: 179.5 ± 8 cm, body mass: 88.6 ± 11 kg) participants, with no resistance training experience within the last three months completed this study. Some participants indicated involvement in other weekly activities, such as walking (*n* = 2) and jogging/running (*n* = 3). The University Institutional Review Board approved this study for Human Subjects. The participants had no known cardiovascular, pulmonary, metabolic, muscular, and/or coronary heart disease. The participants were asked to continue with the same weekly exercise and physical activity schedule but to abstain from exercising the day prior to each testing session. All participants completed a health history questionnaire and signed a written informed consent document before testing.

### 2.3. One Repetition Maximum (1RM) Barbell Bench Press

The flat bench press 1RM strength testing [17] began with a warm-up set of 8–10 reps, using only the barbell (females—15 kg, males—20 kg) as resistance, followed by a 1-min rest. A second warm-up set of 8–10 reps was performed using an estimated 50% of the subject’s 1RM as resistance, followed by another 1-min rest. The third warm-up set was performed at a resistance 4.5–9 kg greater than the previous warm-up set for 3–5 reps, followed by another 1-min rest. The next set was the first test set, using an estimated near maximal (90–95% 1RM) for 2–3 reps followed by a 2-min rest. An additional 4.5–9 kg was added to the resistance from the previous set and 1 repetition was performed. For each subsequent 1RM attempt, 2.2–4.5 kg of resistance was added after each successful repetition, with 2-min rest between each attempt. During each rep, the participant was instructed to keep their feet, glutes and upper back against the bench at all times, failure to do so, was deemed a missed rep. The barbell was to move through a complete range of motion, with the eccentric phase of the lift being controlled down until the bar touched the chest, then, without bouncing the bar off the chest, the concentric phase of the lift was performed to full extension of the arms. Strong verbal encouragement was given for each repetition. These procedures were continued until the subject failed to successfully perform a repetition through the full range of motion, without bouncing the bar off the chest. The resistance of the last successful repetition was considered the participant’s 1RM, with the goal of achieving this within 5 sets. Within our lab, the 1RM bench press test has been shown to be a highly reliable measure, with previous ICC values of 0.99 and no mean difference between test-retest (*p* = 0.052) [15]. The 1RM bench press velocities for each subject were measured by a GymAware (Kinetic Performance, Australia) linear position transducer. The GymAware tether was securely fastened with the supplied Velcro strap, 15–20 cm from the end of the bar, on the participant’s right side.

### 2.4. Bench Throw Test

Five minutes [17] after the 1RM barbell flat bench press was determined, the participants completed a bench throw test. The bench throw test [18] was performed on a Smith machine (LifeFitness, Rosemount, IL, USA), with the participant supine on a flat bench. A weight equal to 35% of the participants’ bench press 1RM was used. The participants were instructed to begin the movement with the arms fully extended and then lower the barbell in a rapid, but controlled (without pulling or allowing the barbell to bounce off the chest) manner, and then immediately move the barbell as fast as possible from the chest. The bar was released on the throw and caught by the participant as the bar descended to the start position. To ensure the safety of the participant, safety pins were placed so that the bar was unable to descend farther than the height the participant’s chest while positioned on the bench. The researchers were also stationed on the end of the bar to aid as a spotter if needed. The participant completed three throws. The bench throw velocities for each participant were measured by a GymAware linear position transducer (Kinetic Performance, Canberra, Australia). The GymAware tether was securely fastened with the supplied Velcro strap, 15–20 cm from the end of the bar, on the participant’s left side.

### 2.5. Training

Each training session began with two warm-up sets. The first warm-up consisted of 10 repetitions, using only the barbell (females—15 kg, males—20 kg) as resistance, followed by a one-minute rest. The second warm-up set consisted of 6 repetitions, utilizing 40–45% of the participant’s 1RM, again followed by a one-minute rest. However, if 40–45% of the participant’s 1RM was lighter than the weight of the bar, the second warm-up set once again used only the bar as resistance. The training session protocol utilized 65% of the participant’s 1RM as resistance for 5 sets of 6 repetitions, with one-minute rest between each set. This volume is based on Prilepin’s Chart, which has previously been used to determine training volume [19]. The participant was instructed to keep their feet, glutes and upper back against the bench during each rep, failure to do so, was deemed a missed rep. During each rep, the participant was instructed to move through a complete range of motion, with the eccentric phase of the lift being controlled down until the bar touched the chest, then, without bouncing the bar off the chest, the concentric phase of the lift to full extension of the arms with maximum effort and velocity. Strong verbal encouragement was given for each repetition.

### 2.6. Statistical Analyses

Statistical analyses consisted of five separate 2 (group: male, female) × 2 (time: pre-test and post-test) mixed model analyses of variance (ANOVAs) and paired samples *t*-tests for 1RM, BP_MV_, BP_PV_, BT_MV_, and BT_PV_. The partial eta squared was used as a measure of effect size. The percent change in 1RM strength ((post-test − pre-test/pre-test) × 100%) for males and females from pre- to post-test was examined with an independent samples *t*-test. An alpha level of *p* ≤ 0.05 was considered statistically significant for all comparisons. All statistical analyses were performed with Statistical Package for the Social Sciences software (v.22.0. IBM SPSS Inc., Chicago, IL, USA).

## 3. Results

**Figure 1:**
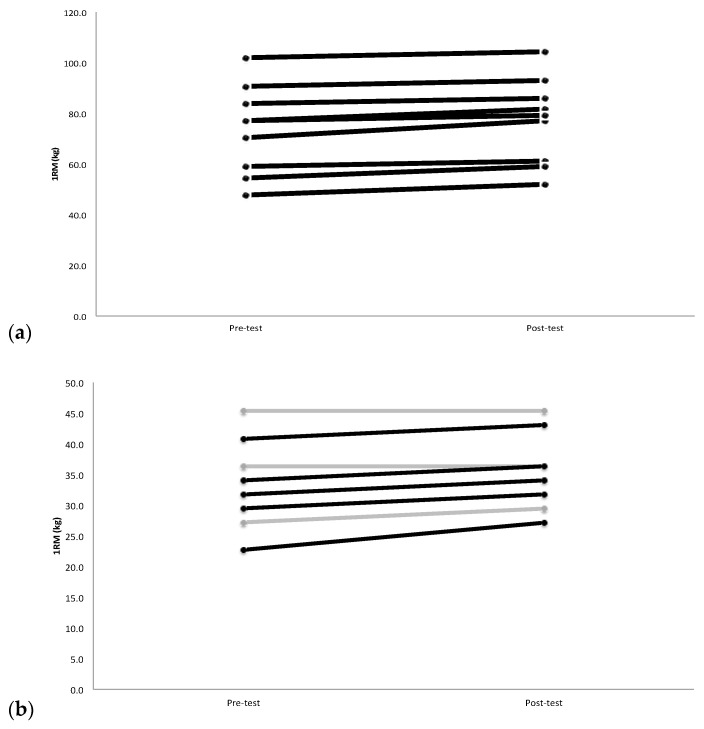
(**a**) Male individual changes in barbell bench press 1 repetition maximum (1RM), from pre-test to post-test. Black lines indicate an increase, where as the grey lines indicate a decrease or no change. *n* = 10; (**b**) Female individual changes in barbell bench press throw mean velocities (BT_MV_), from pre-test to post-test. Black lines indicate an increase, whereas the grey lines indicate a decrease or no change. *n* = 10.

**Figure 2:**
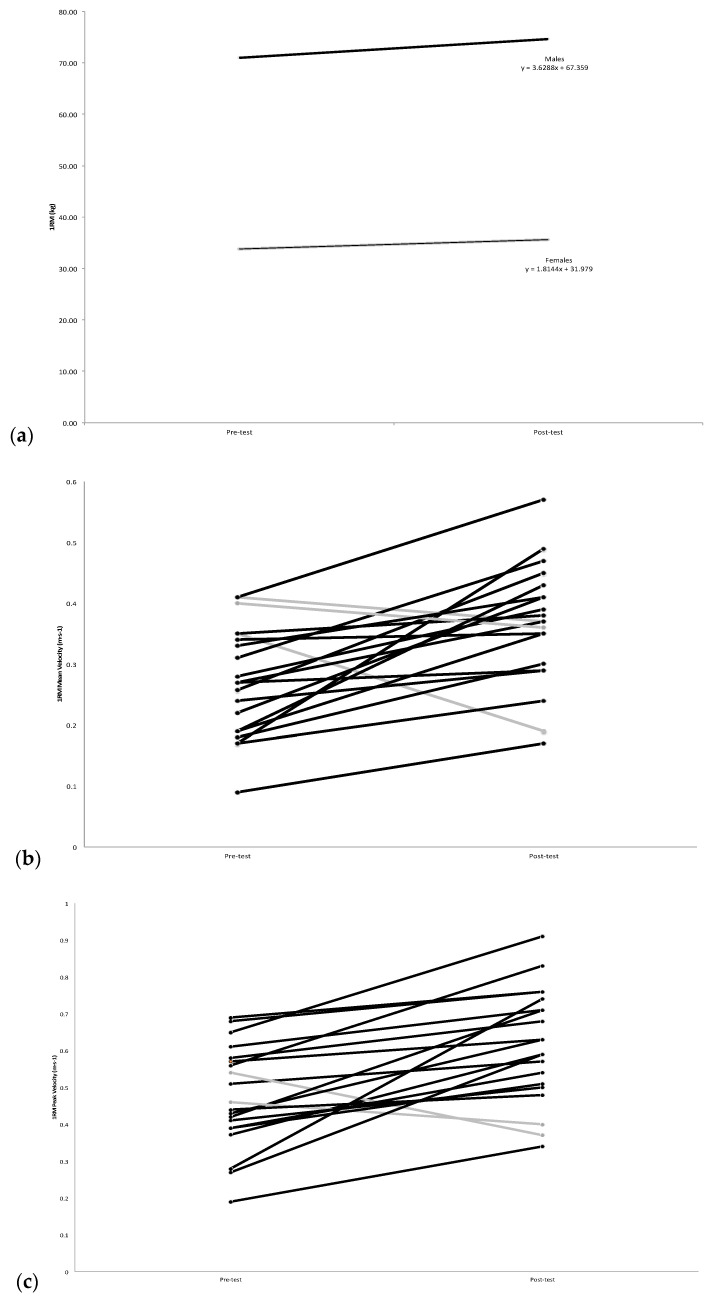
(**a**) Average overall changes in barbell bench press 1 repetition maximum (1RM), from pre-test to post-test. Males *n* = 10 (black line), Females *n* = 10 (grey line); (**b**) Individual changes in 1RM bench press barbell mean velocities (BP_MV_), from pre-test to post-test. Black lines indicate an increase, where as the grey lines indicate a decrease or no change. *n* = 20; (**c**) Individual changes in barbell bench press throw peak velocities (BT_PV_), from pre-test to post-test. Black lines indicate an increase, whereas the grey lines indicate a decrease or no change. *n* = 20.

The 2-way mixed model ANOVA for 1RM strength indicated a significant sex x time interaction (*p* = 0.015, F = 7.194, ηp^2^ = 0.286). There were significant increases in 1RM strength for both males (+3.63 kg, *p* = 0.0001) (Figure 1a) and females (+1.82 kg, *p* = 0.003) (Figure 1b), but the rate of increase for males was greater than females (Figure 2a). However, there was no significant difference between males (5.11%) and females (5.39%) in the percent change of 1RM strength (*p* = 0.82) from pre-test to post-test. The 2-way mixed model ANOVAs indicated no significant sex x time interactions for BP_MV_ (*p* = 0.989), BP_PV_ (*p* = 0.966), BT_MV_ (*p* = 0.735), and BT_PV_ (*p* = 0.886). Table 1 shows the mean (±SD) values (males and females) for pre-test and post-test for 1RM, BP_MV_, BP_PV_, BT_MV_, and BT_PV_. The follow up pairwise comparisons from pre- to post-test (collapsed across sex) showed significant increases for BP_MV_ (*p* = 0.001) (Figure 2b), BP_PV_ (*p* = 0.0001) (Figure 2c), BT_MV_ (*p* = 0.005) and BT_PV_ (*p* = 0.014).

## 4. Discussion

The current findings indicated an increase in strength and barbell velocity for both males and females as a result of VST upper body DCER exercise in untrained subjects. The absolute change in 1RM strength from pre-test to post-test was significantly greater for males (3.63 kg (5.11%)) than for females (1.82 kg (5.39%)), but the percent change was equal (Figure 2a). The greater rate of absolute change, but same percent change, reflected the greater initial 1RM BP strength values for the males compared to the females. Researchers have previously shown a sex difference within the forearm flexors utilizing the VST model, in which the peak torque and average power for males increased 8.4 to 20.2% for concentric isokinetic muscles actions, but no change was observed for females [10]. Based on these findings, the authors suggested 50% of the training-induced performance increases within peak torque of forearm flexion occur within the first three training sessions for males, but not females [10]. In contrast, peak torque increases ranging from 11.5% to 40.2%, depending on the training velocity, were reported for females after isokinetic leg extension VST [11]. Thus, unlike the findings of Traylor et al. [10] for the forearm flexors, the current findings indicated significant increases in upper body strength and barbell velocity for both males and females as a result of DCER VST. The adaptations observed for females in the present study were consistent with those reported for the leg extensors after VST in females [11]. Therefore, the results of the present study in conjunction with those of Traylor et al. [10], Coburn et al. [11], and Prevost et al. [12] indicated sex-, mode-, and muscle group-specific responses to VST.

Overall, there was an increase in 1RM (5.2%), BP_MV_ (32.7%), BP_PV_ (29.8%), BT_MV_ (3.2%), and BT_PV_ (3.2%) for males and females. These strength and velocity adaptations with three days of training were likely not related to skeletal muscle hypertrophy. Moritani [8] suggested the strength gains observed after 2 weeks of training (12 total isotonic training sessions) were largely due to neural factors. Possible mechanisms behind these performance increases may be related to neuromuscular adaptations such as an increased motor unit firing rate of the active muscle, recruitment of more motor units, or an increase in agonist muscle activation and/or decrease in antagonist muscle coactivation [11,16]. However, previous studies have reported no increased agonist muscle activation after 2 days of isokinetic training [20]. Akima [21] suggested strength gains after short training periods (9 total isokinetic training sessions) were due to increases in muscle contractile activity. Electromyography (EMG) has been used to further understand the possible neuromuscular responses of skeletal muscle, with the amplitude of the EMG signal reflecting global motor unit activation and the mean power frequency (MPF) reflecting the conduction velocity of the action potential along the sarcolemma [20,22]. The use of mechanomyography (MMG) provides the mechanical counterpart to the motor unit electrical activity measured by EMG [20,22]. The MMG amplitude reflects motor unit recruitment, and the MMG frequency domain provides qualitative information regarding the global firing rate of the unfused activate motor units [20,22]. Utilizing EMG and MMG signals, researchers have provided evidence that the strength increases observed from VST are potentially due to increases in motor unit firing rate of the active muscle, rather than increases in motor unit recruitment [16]. Future studies should examine changes in the amplitude and frequency domains of the EMG and MMG signals to further elucidate the neuromuscular adaptations that result from DCER VST.

The current findings indicated increases in strength and barbell velocity for males and females as a result of upper body DCER VST. These findings, in conjunction with those of others [10,11,12], indicated there are sex-, mode-, and muscle-specific adaptations to VST. Thus, future studies should examine the effects of lower body DCER VST to determine if the strength and barbell velocity adaptations observed for the upper body for males and females in this study are also present within the lower body. One limitation of our findings is the lack of a control group; however, the results of previous studies from our lab have shown the 1RM BP test to be a highly reliable test [15]. Another limitation is with this study being the first study to our knowledge to examine the effects of upper body DCER VST training on sex; there were no other references available to specifically compare our findings. The VST model has a number of clinical applications for rehabilitation after injury or surgery. Rehabilitation programs, however, often focus only on the injured limb. Thus, it is important to determine what effects unilateral and bilateral VST may have on the bilateral deficit in both upper and lower limbs. In addition, the examination of the EMG and MMG signals pre- and post-VST may allow researchers to better understand the neuromuscular adaptations responsible for the early-phase strength and performance adaptations.

## Figures and Tables

**Table 1:** Mean ± standard deviation (SD) pre-test and post-test values for male and female participant’s barbell bench press 1 repetition maximum (1RM), the mean barbell velocity from the subject’s 1RM (BP_MV_), the peak barbell velocity from the subject’s 1RM (BP_PV_), the mean velocity of the barbell bench press throw test (BT_MV_), and the peak velocity of the barbell bench press throw test (BT_PV_).

	Males (*n* = 10)		Females (*n* = 10)	
	Pre-test	Post-test	Pre-test	Post-test
**1RM (kg)**	70.99 ± 18.58	74.62 ± 17.84	33.79 ± 5.61	35.61 ± 5.56
**BP_MV_ (m·s^−1^)**	0.28 ± 0.12	0.37 ± 0.12	0.27 ± 0.06	0.36 ± 0.08
**BP_PV_ (m·s^−1^)**	0.49 ± 0.16	0.63 ± 0.16	0.45 ± 0.12	0.59 ± 0.15
**BT_MV_ (m·s^−1^)**	0.89 ± 0.06	0.91 ± 0.05	0.69 ± 0.07	0.72 ± 0.05
**BT_PV_ (m·s^−1^)**	1.59 ± 0.1	1.64 ± 0.12	1.22 ± 0.11	1.26 ± 0.09

There was a significant (*p* < 0.05) increase from pre-test to post-test in all variables collapsed across gender.

## References

[1] Housh T.J., Housh D.J., Weir J.P., Weir L.L. (1996). Effects of unilateral concentric-only dynamic constant external resistance training. Int. J. Sports Med..

[2] Gosran P., Myklestad D., Raastad T. (2003). The influence of volume of exercise on early adaptations to strength training. J. Strength Cond. Res..

[3] Kraemer W.J., Patton J.F., Gordon S.E., Harman E.A., Deschenes M.R., Reynolds K., Newton R.U., Triplett N.T., Dziados J.E. (1995). Compatibility of high-intensity strength and endurance training on hormonal and skeletal muscle adaptations. J. Appl. Physiol..

[4] Grgic J., Schoenfeld B.J., Davies T.B., Lazinica B., Krieger J.W., Pedisic Z. (2018). Effect of resistance training frequency on gains in muscular strength: A systematic review and meta-analysis. Sports Med..

[5] Aagaard P., Simonsen E.B., Andersen J.L., Magnusson P., Dyhre-Poulsen P. (2002). Increased rate of force development and neural drive of human skeletal muscle following resistance training. J. Appl. Physiol..

[6] Staron R.S., Karapondo D.L., Kraemer W.J., Fry A.C., Gordon S.E., Falkel J.E., Hikida R.S. (1994). Skeletal muscle adaptations during early phase of heavy-resistance training in men and women. J. Appl. Physiol..

[7] Østerås H., Helgerud J., Hoff J. (2002). Maximal strength-training effects on force-velocity and force-power relationships explain increases in aerobic performance in humans. Eur. J. Appl. Physiol..

[8] Moritani T., Devries H.A. (1979). Neural factors versus hypertrophy in the time course of muscle strength gain. Am. J. Phys. Med..

[9] Jones D.A., Rutherford O.M. (1987). Human muscle strength training: The effects of three different regimens and the nature of the resultant changes. J. Physiol..

[10] Traylor D.A., Housh T.J., Lewis R.W., Bergstrom H.C., Cochrane K.C., Jenkins N.D., Cramer J.T. (2014). The effects of gender and very short-term resistance training on peak torque, average power and neuromuscular responses of the forearm flexors. Isokinet. Exerc. Sci..

[11] Coburn J., Housh T.J., Malek M.H., Weir J., Cramer J.T., Beck T.W., Johnson G.O. (2006). Neuromuscular responses to three days of velocity-specific isokinetic training. J. Strength Cond. Res..

[12] Prevost M., Nelson A.G., Maraj B.K.V. (1999). The effect of two days of velocity-specific isokinetic training on torque production. J. Strength Cond Res..

[13] Costa P.B., Herda T.J., Walter A.A., Valdez A.M., Cramer J.T. (2013). Effects of short-term resistance training and subsequent detraining on the electromechanical delay. Muscle Nerve.

[14] Costa P.B., Herda T.J., Herda A.A., Cramer J.T. (2016). Effects of Short-Term Dynamic Constant External Resistance Training and Subsequent Detraining on Strength of the Trained and Untrained Limbs: A Randomized Trial. Sports.

[15] Byrd M.T., Bergstrom H.C. (2018). Effects of Very Short-Term Dynamic Constant External Resistance Exercise on Strength and Barbell Velocity in Untrained Individuals. Int. J. Exerc. Sci..

[16] Cramer J.T., Stout J.R., Culbertson J.Y., Egan A.D. (2007). Effects of creatine supplementation and three days of resistance training on muscle strength, power output, and neuromuscular function. J. Strength Cond. Res..

[17] Baechle T.R. (2008). Essentials of Strength and Conditioning.

[18] Thomas G.A., Kraemer W.J., Spiering B.A., Volek J.S. (2007). Maximal power at different percentages of one repetition maximum: Influence of resistance and gender. J. Strength Cond. Res..

[19] Hammer E. (2009). Preseason training for college baseball. Strength Cond. J..

[20] Beck T., Housh T.J., Johnson G.O., Weir J., Cramer J.T., Coburn J.W., Malek M.H., Mielke M. (2007). Effects of two days of isokinetic training on strength and electromyographic amplitude in the agonist and antagonist muscles. J. Strength Cond. Res..

[21] Akima H., Takahashi H., Kuno S.Y., Masuda K., Masuda T., Shi-mojo H., Anno I., Itai Y., Katsuta S. (1999). Early phase adaptations of muscle use and strength to isokinetic training. Med. Sci. Sports Exerc..

[22] Beck T.W., Housh T.J., Cramer J.T., Weir J.P., Johnson G.O., Coburn J.W., Malek M.H., Mielke M. (2005). Mechanomyographic amplitude and frequency responses during dynamic muscle actions: A comprehensive review. Biomed. Eng. Online.

